# Editorial: Advanced Strategies for the Recognition, Enrichment, and Detection of Low Abundance Target Bioanalytes

**DOI:** 10.3389/fbioe.2022.957878

**Published:** 2022-08-17

**Authors:** Jian Zhang, Jie Jayne Wu, Li Chen

**Affiliations:** ^1^ College of Electrical and Electronic Engineering, Wenzhou University, Wenzhou, China; ^2^ Department of Electrical Engineering and Computer Science, The University of Tennessee, Knoxville, TN, United States; ^3^ College of Photoelectric Engineering, Chongqing University, Chongqing, China

**Keywords:** low abundance, target bioanalytes, recognition, enrichment, detection

Biosensors and bioassays provide important analysis capabilities for biological, medical, and environmental applications. Owing to active and creative exploration of emerging materials, devices, and methods, there have been great advances in biosensors and bioassays, making detection more sensitive, rapid, accessible, and intelligent. For biodetection, especially for low abundance, strategies for target recognition, enrichment, and detection are of vital importance.

The goal of this Research Topic is to provide a forum sharing recent research and novel ideas on related techniques. We have collected eight papers focused on biosensors for target or event detection: three research articles, two opinion articles, and three reviews. The techniques include microfluidics, electrochemistry, CRISPR, emerging materials, and so on.

In terms of research papers, Zhu et al. report a microfluidic device integrated with an electrical impedance spectroscopy (EIS) biosensor to perform *in-situ* impedance measurement of yeast proliferation in single-cell resolution. Hydraulic shear force is utilized to detach a daughter cell from its mother cell, and time-lapse impedance measurement is performed to monitor the cellular process. The main highlight of this work is a combined sensor achieving *in-situ* and real-time monitoring of a dynamic event for single-cell reproduction, which uses simple EIS instead of image observation, saving hardware resources for image recording. Meanwhile, the capacity to monitor for longer than several hours still needs to be improved.

Salvador et al. present the development of a fluorescent microfluidic device based on an antibody microarray for therapeutic drug monitoring of acenocoumarol (ACL). A fully integrated microfluidic system is realized using a specific antibody for ACL on the glass slide in a microarray chip, with a fluorescent label for target binding. A limit of detection (LOD) of 1.23 nM is achieved in human plasma. As a proof-of-concept point-of-care device, this system is automatic and sensitive, although the exact turnaround time is not mentioned. The selectivity also needs to be demonstrated for situations such as the presence of other molecules similar to ACL existing in the blood.

Yuan et al. have developed a one-step electropolymerized biomimetic polypyrrole membrane-based electrochemical sensor for selective detection of valproate (VPA). The critical point of this strategy is to fabricate molecularly imprinted polymer (MIP) with simple one-step electropolymerization and employ the MIP/gate effect to realize sensitive concentration-electrical signal response. This sensor has a LOD of 17.48 μM and good selectivity against five drugs in combination therapy with VPA. This work furthers a step toward conveniently controlling VPA concentrations for patients, and the main shortfall is the lack of confirmatory tests in practical blood or serum, which is a much more complex matrix than deionized water.

For opinions, Jiang et al. introduce the working modes and applications of biosensors for point mutation detection, including the biosensors for RNA mutation rate detection of SARS-CoV-2. As an alternative to traditional methods, biosensors for gene mutation detection are fast, low-cost, and suitable for large-scale applications. Using ssDNA probes for hybridizing with the point mutations, gene sequencing, and PCR-based methods become unnecessary. Meanwhile, versatile signal conversion approaches enable various sensitive sensors. As a result, the LOD for point mutation can be as low as 0.5 fM. ssDNA is commonly designed as an oligonucleotide for biomolecule recognition, while the basic function of hybridization promotes the detection of point mutation.

Jiang et al. describe a possibility for the application of artificial intelligence-based triboelectric nanogenerators (AI-TENGs) in biosensor development. The mechanism of triboelectric nanogenerators and their combination with artificial intelligence are introduced. Representative research on AI-TENG for biomolecules sensing and other biotechnology is then highlighted. TENG as an energy collection and conversion technique is of great value for portable biosensors needing a power supply, while more investigations might be needed on the integration of AI and TENG for biosensor development.

In terms of the reviews, Li et al. review recent advances in metal-organic framework (MOF)-based electrochemical biosensing applications. The main content includes the synthesis and modification of MOFs for electroactive materials and emerging biosensing applications of these MOF materials. The emphatically introduced applications are small-biomolecule sensing, biomacromolecule sensing, and pathogenic cell sensing. The specific improvement to figures of merit by MOFs, such as LOD, is not presented in this paper, and oxidative degradation in certain solutions might be considered when MOFs are applied in electrochemical sensors.

Liu et al. discuss nanomaterial-based immunocapture platforms for recognition, isolation, and detection of circulating tumor cells (CTCs). Three parts are outlined: design principles for immunoaffinity nanomaterials, nanomaterial-based platforms for CTC immunocapture and release, and recent advances in single-cell release and analysis for CTCs. Nanomaterials of different shapes, sizes, and structures are introduced, showing their good performance for agent linking and CTC isolation and detection. In this article, capture efficiency is highlighted as an important figure of merit. Although the overall detection time of most mentioned techniques is not introduced, this review remains a comprehensive review of CTC separation and detection.

Chen et al. have created an overview of the recent development of clustered regularly interspaced short palindromic repeats (CRISPR)-based biosensing techniques and their integration with microfluidic platforms. A representative microfluidic CRISPR sensor achieves a LOD of 20 pfu/ml of purified Ebola RNA within 5 min, and most similar sensors are all accompanied by high sensitivity and fast responses.

Working with the two powerful techniques, these biosensors, being applied in nucleic acid-based diagnostics, protein tests, metal ion monitoring, and protein/small molecule interactions screening, have great advantages for point-of-care testing of various bio-targets with low abundance.

In summary, the articles collected in this Research Topic include various emerging materials, methods, strategies, and platforms promoting technological innovation for recognition, enrichment, and detection of low abundance target bioanalytes. In these articles, microfluidic devices are most utilized for target collection and enrichment, while electrochemical biosensors are widely adopted transducers for signal conversion. Advanced and interdisciplinary fields such as CRISPR, MOF, and AI-TENG are represented in these articles.

For the goal of trace bio-target analysis, the decisive techniques are target enrichment and sensing mechanism, possibly including signal amplification. Efficient target enrichment and sensitive signal transformation remain critical challenges to be solved, and efforts are being made to seek advanced strategies besides the methods mentioned in this Research Topic. As a promising strategy, AC electrokinetics is demonstrated to be a simple and versatile approach for rapid bio-target enrichment (Salari and Thompson, 2018). Meanwhile, researchers have developed an ultrasensitive sensing mode by monitoring the solid-liquid interfacial capacitance of an electrode array. Combining the two mechanisms, the LOD level of 0.1 fM (Zhang et al., 2021), fg/mL (Qi et al., 2022), or 10 CFU/ml (Zhang et al., 2020) is achieved when ions, proteins, or bacteria are detected with the turnaround time less than 1 min. With technological advances being continuously on the horizon, more sensitive, convenient, rapid, and intelligent detection is expected to emerge in the future.
